# Fecal Microbiota Transplantation Is Effective in Relieving Visceral Hypersensitivity in a Postinfectious Model

**DOI:** 10.1155/2018/3860743

**Published:** 2018-01-30

**Authors:** Tao Bai, Lei Zhang, Huan Wang, Wei Qian, Jun Song, Xiaohua Hou

**Affiliations:** Division of Gastroenterology, Union Hospital, Tongji Medical College, Huazhong University of Science and Technology, Wuhan, Hubei 430022, China

## Abstract

**Aim:**

To investigate the effect of fecal microbiota transplantation on visceral hypersensitivity compared with* Bifidobacterium longum*.

**Methods:**

Mice visceral hypersensitivity was induced by* Trichinella spiralis*. After 8 weeks, they were divided into three groups (controls,* Bifidobacterium longum*, and fecal microbiota transplantation) and were daily treated by gavage with 0.2 ml PBS,* Bifidobacterium longum* HB55020, or fecal microbiota for 7 days. Visceral hypersensitivity was tested with abdominal withdrawal reflex. Permeability of colon epithelium was assessed with Ussing chamber.

**Results:**

After administration of* Bifidobacterium longum*, compared with mice in postinfectious group, mice had higher pain threshold (*p* < 0.05). After administration of fecal microbiota, compared with mice in postinfectious group, mice had higher pain threshold (*p* < 0.05). Fecal microbiota transplantation was as effective as* Bifidobacterium* in relieving visceral hypersensitivity. Administration of* Bifidobacterium longum* or fecal microbiota transplantation improved colon epithelium permeability. Expression of occluding-1 was increased.

**Conclusion:**

Manipulation of microbiota is effective in relieving visceral hypersensitivity. Fecal microbiota transplantation is as effective as* Bifidobacterium longum* administration.

## 1. Introduction

Abdominal pain and/or discomfort are common symptoms in various disease situations [1–3] and the severity of abdominal symptoms significantly influences patients' quality of life and psychological disturbances [4, 5]. Visceral hypersensitivity plays a key role in inducing abdominal symptoms such as functional abdominal pain syndrome, irritable bowel syndrome, and ulcerative colitis. However, the mechanism remains unclear and clinical management of visceral hypersensitivity is limited and unsatisfactory [6].

GI microbiota contains microorganisms in number of more than ten times the body's own cells [7]. Intestinal microorganisms and their metabolism of nutrients are important in health maintenance and disease development. Recently, studies on GI microbiota suggested that microbiota as well as its metabolites is involved in pathophysiology of visceral hypersensitivity [8]. Exciting preclinical outcomes were reported as for efficacy of microbiota management of visceral pain [9–11]. On the other hand, a systematic review indicated that probiotics were effective overall for management of lower GI symptoms [12]. However, the administration varies as for probiotics, dose, frequency, and duration. There is still a long way to go to optimize the therapeutic choices.

It is worth noting that the stability of GI microenvironment is influenced not only by bacteria, but also by other microorganisms and substances including metabolism and other luminal contents [13–15]. It is rather like a system than a single composition. Recently a published preliminary study showed that transfer of sterile filtrates from donor stool can eliminate symptoms. That indicates that bacterial components, metabolites, and/or bacteriophages are also important [16]. Therefore, fecal microbiota transplantation (FMT) could be a better choice to relieve visceral hypersensitivity.

This study aims to investigate the effect of FMT on the elimination of visceral hypersensitivity, compared with* Bifidobacterium longum*, and to detect the potential mechanism in a postinfectious animal model.

## 2. Materials and Methods

### 2.1. Animals

Male NIH mice (6–8 weeks old) were ordered from Medical Animal Laboratory Center of Guangdong. All mice were raised under the specific pathogen-free condition. All the experimental procedures were approved by Ethics Committee of Tongji Medical College.

### 2.2. Visceral Hypersensitivity Model

An animal model of visceral hypersensitivity was performed as described previously [17]. The model was induced by* Trichinella spiralis*, which was obtained from the Department of Parasitology, Huazhong University of Science and Technology. The colony of* Trichinella spiralis* was maintained in muscle tissue by infecting the Sprague-Dawley rats. We obtain the larvae from infected rats with the methods described by Castro and Fairbairn [18]. We counted* Trichinella spiralis* larvae under microscope and each mouse was infected by gavage with 350 larvae in 0.2 ml of phosphate-buffered saline (PBS).

### 2.3. Probiotics and FMT

The live strain of* Bifidobacterium longum* HB55020 (1.66 × 10^12^ CFU/g) was obtained from Hubei Center of Industrial Culture Collection and Research, HBCC. The strain was mixed with glucose and converted to freeze-dried powder. The mixed powder was packed in sealed bags of 2 g and stored at −20°C for further use. The viable bacterial count was credible and was calculated by culture and colony counting method after dilution. Fresh fecal pellets of 3-4 uninfected mice were collected. We pooled them together and weighed them. Then the fecal pellets were placed in 0.25–1.0 ml of sterilized PBS and the volume was adjusted to give 120 mg feces per milliliter. The fecal pellets were mashed with sterile wooden toothpicks and then vortexed at maximum speed for 1 min. The fecal matter was centrifuged for 3 min at 800 ×g, and the supernatant was used for FMT [19]. Fresh fecal supernatant was prepared every day during the treatment with the same protocol.* T. spiralis*-infected mice after 8 weeks were divided into three groups (controls,* Bifidobacterium longum* group, and FMT group). Controls were daily treated by gavage with 0.2 ml PBS for 7 days. Mice in* Bifidobacterium longum* group were daily treated with* Bifidobacterium longum* HB55020 (2 × 10^9^ CFU/d) for 7 days. Mice in FMT group were treated with 0.2 ml fecal microbiota for 7 days.

### 2.4. Study Design

Thirty mice were randomly divided into four groups: control (*n* = 6), postinfection group (8-week postinfection) (*n* = 8),* Bifidobacterium longum* group (*n* = 8), and FMT group (*n* = 8). We tested visceral hypersensitivity to evaluate the success of the animal model. We assessed the permeability of colon epithelium with Ussing chamber. Real-time polymerase chain reaction was performed to compare the mRNA transcription of tight junction protein. Abdominal withdrawal reflex to colorectal distention [20] was recorded to assess visceral sensitivity. Colorectal distention was performed as our former study [20]. Abdominal withdrawal reflex was recorded during plastic balloon inflation to 20, 40, 60, and 80 mmHg. Threshold intensity of colorectal distention was recorded when the stimulus intensity evoked a visually identifiable contraction of the abdominal wall. Colorectal distention was performed in mice for 20 seconds every 4 minutes. Two investigators observed the abdominal withdrawal reflex independently, and balloon inflation was done for five times to achieve an accurate result.

### 2.5. Permeability Assessment

The colon was quickly removed and flushed with ice-cold Krebs solution (121 mM NaCl, 25 mM NaHCO3, 3.8 mM KCl, 1 mM KH2PO4, 1.2 mM CaCl2, 1.2 mM MgSO4, and 11.1 mM glucose). The external muscle and myenteric plexus were stripped off. Each piece was placed in an Ussing chamber (Physiology Instruments, Santiago, CA), and both sides of the chamber were filled with 5 ml Krebs solution, oxygenated, and maintained at 37°C throughout the experiment. The spontaneous potential difference and short circuit current (Isc) were recorded in the Ussing chambers after a 30-min equilibration period. Transepithelial electrical resistance was calculated with Acquire and Analyze 2.3 software.

Three milliliters of FITC-dextran (FD4, 1 mg/ml, Sigma-Aldrich, USA) was added to the mucosal side, and an equivalent volume of Krebs solution was added to the other side of each chamber. At 30-min intervals, 100-*μ*l samples were collected and transferred to 96-well plates in duplicate. Krebs solution (200 *μ*l) was added to the Ussing chambers after fluid collection to equalize the volumes. The FD4 flux of each sample was examined at 520 nm with a Fluorescence Microplate Reader (Molecular Devices, USA). The FD4 concentration was determined based on the standard curves as described previously [21]. The permeability of each piece of tissue was presented as the calculated flux of FD4 over a 30–60 min period.

### 2.6. Real-Time Reverse-Transcription Polymerase Chain Reaction

Total cellular RNA was extracted from intestine segments with Trizol (Gibco) using a standard method according to the manufacturer. An aliquot (*μ*g) of RNA was reverse-transcribed into first-strand complementary DNA (cDNA) as the manufacture's recommendation of Takara Kits (Takara Biomedicals, Japan). Real-time reverse-transcription polymerase chain reaction was carried out with a light cycler using DNA-binding dye SYBR Green for detection of PCR products. The reaction mixture contained 5 *μ*l of SYBR Green, 3 *μ*l RNAase free water, 1 *μ*l primer, and 1 *μ*l cDNA to give a final reaction volume of 10 *μ*l. The sequences of primers were shown as follows: (5′-3′). *β*-actin, TGTTACCAACTGGGACGACA for forward and CTGGGTCATCTTTTCACGGT for reverse; claudin-1, TATGACCCCTTGACCCCCAT for forward and TTGTTTTCCGGGGACAGGAG for reverse; ZO-1, GCTTTAGCGAACAGAAGGAGC for forward and TTCATTTTTCCGAGACTTCACCA for reverse; occludin-1, TGAAAGTCCACCTCCTTACAGA for forward and CCGGATAAAAAGAGTACGCTGG for reverse.

### 2.7. Statistical Analysis

Abdominal withdrawal reflex scores at each pressure of colorectal distention between control and model groups were expressed as median (interquartile range) and compared with Kruskal-Wallis test. A Wilcoxon rank sum test was performed with a Bonferroni correction at 0.05/3 to correct for multiple comparisons. Other data were expressed as means ± standard errors and compared with *t*-test or variance analysis. Statistical analyses were performed using R 2.11.1 software. GraphPad Prism 5 software (GraphPad, USA) was used for all graph creation. A *p* value of < 0.05 was considered significant.

## 3. Results

### 3.1. Assessment of Visceral Sensitivity after* Trichinella* Infection

**Figure 1:**
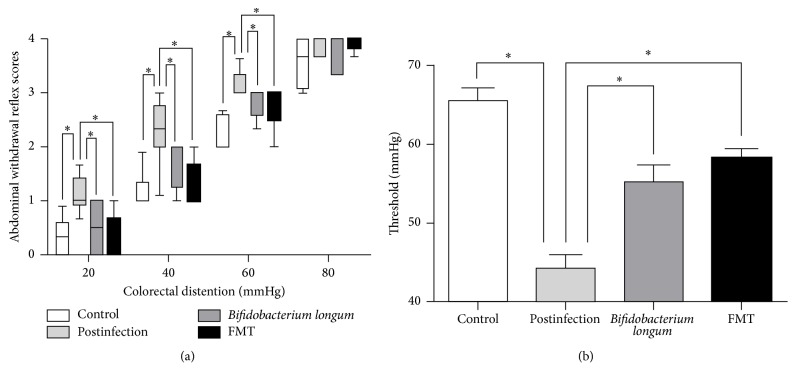
Assessment of visceral sensation. (a) Box plot of the abdominal withdrawal reflex (AWR) scores. The lines in the boxes represent the medians, and the lines at the ends of the boxes represent the 25th and 75th percentiles. The error bars denote the 5th and 95th percentiles. (b) The threshold of colorectal distention (CRD) intensities that evoked abdominal contractions in the mice. The bar graphs are presented as the means ± SE; *n* ≥ 6 mice per group. Postinfection: PBS administration after infection.* Bifidobacterium longum*:* Bifidobacterium longum* administration after infection. FMT: fecal microbiota transplantation after infection. ^*∗*^*p* < 0.05.

All mice treated with* Trichinella* were successfully infected as shown in Figure S1. Compared with uninfected mice, postinfectious mice had higher abdominal withdrawal reflex scores for intensities of 20 mmHg (*p* < 0.05), 40 mmHg (*p* < 0.05), and 60 mmHg (*p* < 0.05) and had significantly lower pain threshold (*p* < 0.05) (Figure 1). These results indicated that after being infected for 8 weeks, mice have visceral hypersensitivity.

### 3.2. *Bifidobacterium longum* and FMT Extenuated Visceral Hypersensitivity

As shown in Figure 1, after administration of* Bifidobacterium longum*, compared with postinfectious group, mice had higher pain threshold (*p* < 0.05) and lower abdominal withdrawal reflex scores for intensities of 20 mmHg (*p* < 0.05), 40 mmHg (*p* < 0.05), and 60 mmHg (*p* < 0.05). After administration of fecal microbiota, compared with postinfectious group, mice had higher pain threshold (*p* < 0.05) and lower abdominal withdrawal reflex scores for intensities of 20 mmHg (*p* < 0.05), 40 mmHg (*p* < 0.05), and 60 mmHg (*p* < 0.05). No significant differences in abdominal withdrawal reflex scores and pain threshold were detected between* Bifidobacterium longum* group and FMT group.

### 3.3. *Bifidobacterium longum* and FMT Potentially Extenuated the Increased Mucosal Permeability

**Figure 2:**
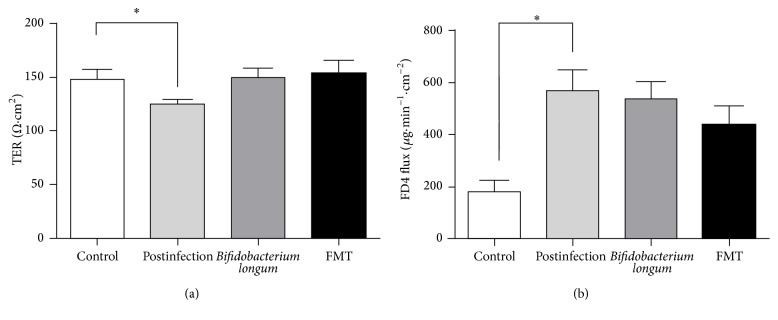
Mucosal permeability increases after infection. (a) The transepithelial electrical resistance (TERs) of colon epithelium. (b) The FD4 fluxes of the four groups. All data are presented as the means ± SE. Postinfection: PBS administration after infection.* Bifidobacterium longum*:* Bifidobacterium longum* administration after infection. FMT: fecal microbiota transplantation after infection. ^*∗*^*p* < 0.05.

Mucosal permeability was tested by Ussing chamber system to examine transepithelial electrical resistance (TER) and FD4 flux (Figure 2). Compared with controls, mice in postinfectious group had lower TER (*p* < 0.05) and higher FD4 influx (*p* < 0.05). This indicated that mice in postinfectious group have higher permeability, and the increased permeability might play a role in visceral hypersensitivity.

After administration of* Bifidobacterium longum* and fecal microbiota, colonic mucosal permeability was assessed. There were potentially significant differences in permeability among the three groups as for TER (*p* = 0.08 for ANOVA). We compared the TER between* Bifidobacterium longum* or fecal microbiota and postinfectious mice and found that* Bifidobacterium longum* potentially extenuated the increased permeability (*p* = 0.08 for *t*-test) and FMT extenuated it significantly (*p* < 0.05 for *t*-test). Although the mean of FD4 influx suggested extenuation after administration of* Bifidobacterium longum* and fecal microbiota, no significant differences were detected.

### 3.4. *Bifidobacterium longum* and Fecal Microbiota Affected the Expression of Tight Junctions

**Figure 3:**
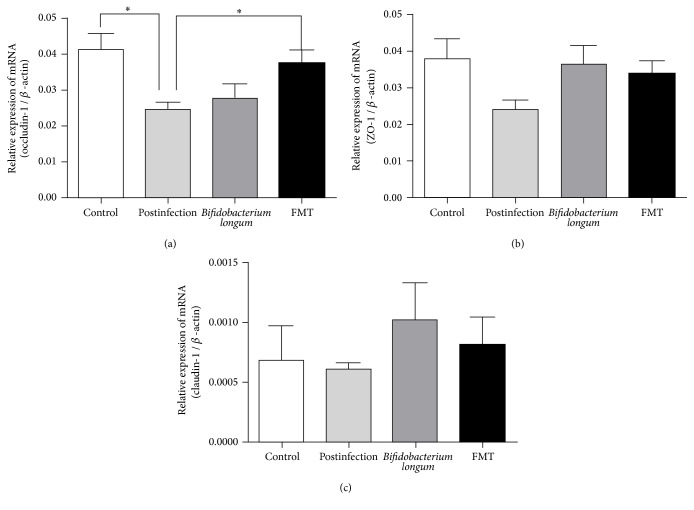
Expression of the tight junction mRNA in colon. (a) Expression of the occluding-1 mRNA. (b) Expression of the ZO-1 mRNA. (c) Expression of claudin-1. All data are presented as the means ± SE. Postinfection: PBS administration after infection.* Bifidobacterium longum*:* Bifidobacterium longum* administration after infection. FMT: fecal microbiota transplantation after infection. ^*∗*^*p* < 0.05.

We further test the mRNA expression of main tight junctions (Figure 3). Compared with controls, postinfectious mice had significant lower mRNA expression of occluding-1 (*p* < 0.05) and potentially significant lower expression of ZO-1 (*p* = 0.06). There were no significant differences as for mRNA expression of claudin-1.

After administration of* Bifidobacterium longum* or fecal microbiota, there were significant differences compared with postinfectious mice (*p* < 0.05 for ANOVA) as for expression of occluding-1. The differences between FMT group and postinfectious group were significant (*p* < 0.05), and the differences between FMT and* Bifidobacterium longum* were not significant. As for ZO-1, there were potential differences among* Bifidobacterium longum* group, FMT group, and postinfectious group (*p* = 0.09 for ANOVA). Compared with postinfectious mice,* Bifidobacterium longum* significantly increases the expression of ZO-1 (*p* < 0.05).

## 4. Discussion

This study indicated that FMT could relieve postinfectious visceral hypersensitivity as effectively as* Bifidobacterium longum* administration. This effect is related to changes of permeability mediated by tight junction. The results highlight the potential optimized choice for clinical management of visceral hypersensitivity.

Microbiota proved to be involved in pathogenesis of visceral pain. It is recently reported that visceral hypersensitivity could be transferred by FMT to the rats from irritable bowel syndrome patients [22]. Clinical studies have found that probiotics administration was overall effective in relieving visceral pain or discomfort [23]. Our study performs an optimized choice, which is directly comparing the efficacy of FMT with that of a single probiotic in relieving visceral hypersensitivity. As for composition of FMT, there are other microorganisms as well as substances that could be effective during the regulation [24]. Compared with a single probiotic, FMT is more like a comprehensive system including mixed bacteria, other microorganisms, metabolism, and other soluble composition. Therefore, it could be described as a complex natural symbiotic system with further potential utility.

There may be several pathways to regulate the visceral hypersensitivity. Firstly, some bacteria could be involved in the regulation of visceral sense.* L. acidophilus* was indicated to upregulate *μ*-opioid and cannabinoid receptors in vitro and in vivo [11]. L.* paracasei* reduced abdominal pain and mucosal inflammation [10]. Secondly, as for metabolism, they can directly influence the nociception and the barrier function [8]. Besides, many metabolites could directly regulate intestinal function such as endogenous vitamins, short chain fatty acids, and neurotransmitters such as serotonin and gamma-aminobutyric acid [13–15]. Barrier function was important in sense of luminal information and played a central role in microbiota mediated visceral sensitivity regulation. Previous studies found the disruption of intestinal barrier function in disease condition which was indicated as a vicious circle [25]. Improvement of barrier function is accordingly of vital importance. Our study finds that permeability was improved by FMT,  which was found to be as effective as* Bifidobacterium longum*. This result indicated that fecal microbiota transplantation is effective in relieving visceral hypersensitivity and could break in the vicious circle by retrieve barrier function disruption.

Preclinical evidence of microbiota manipulation to relieve visceral hypersensitivity mainly comes from stress induction [6]. Therefore, changes in the visceral sensitivity may be secondary to the stressors. Our study used a postinfectious animal model to imitate the change of visceral sensitivity, which could provide evidence as for efficacy of microbiota manipulation to visceral hypersensitivity. Besides, limited studies compare the efficacy of probiotics with FMT, and this study gives direct evidence. However, the dose are referenced from published studies; we are not sure whether there is a dose-response effect. Therefore, better effect could be derived from higher dose. Since fecal microbiota mixture of complex ingredients and many compositions could take part in the regulation,  balance of dose between FMT and probiotics is unachievable. In spite of the fact that direct comparison shows similar effect of microbiota transplantation and gives an optional and optimized choice for preclinical management of visceral hypersensitivity, further clinical studies on efficacy and safety are needed.

In summary, this study finds that FMT is as effective as* Bifidobacterium longum* in relieving visceral hypersensitivity. Occludin-1 mediated permeability could play an important role in regulation of visceral sensitivity.

## Supplementary Materials

Supplementary Materials Figure S1: All mice treated with* Trichinella* were successfully infected by attached preparation of diaphragm (×100).
