# Supplementary figures and images for: Fecal Microbiota Transplantation Is Effective in Relieving Visceral Hypersensitivity in a Postinfectious Model
Source: Biomed Res Int. 2018 Jan 30;2018:3860743. doi: 10.1155/2018/3860743 (PMC5833243; doi:10.1155/2018/3860743)

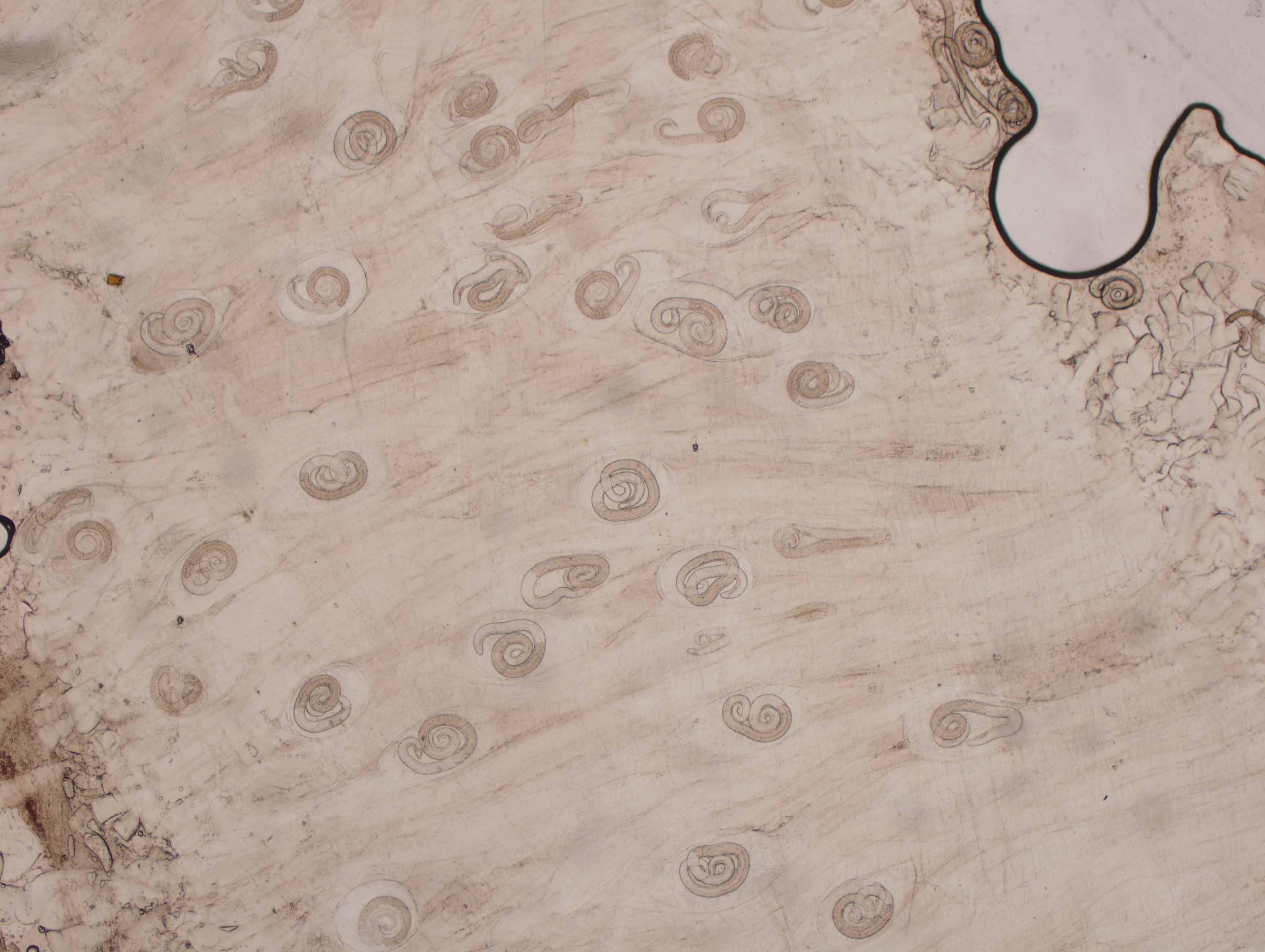

Supplement: Supplementary Materials — Figure S1: All mice treated with Trichinella were successfully infected by attached preparation of diaphragm (×100). [file 3860743.f1.jpg]
